# Molecular Genetic Screening in Patients With ACE Inhibitor/Angiotensin Receptor Blocker-Induced Angioedema to Explore the Role of Hereditary Angioedema Genes

**DOI:** 10.3389/fgene.2022.914376

**Published:** 2022-07-18

**Authors:** Carina M. Mathey, Carlo Maj, Annika B. Scheer, Julia Fazaal, Bettina Wedi, Dorothea Wieczorek, Philipp M. Amann, Harald Löffler, Lukas Koch, Clemens Schöffl, Heinrich Dickel, Nomun Ganjuur, Thorsten Hornung, Susann Forkel, Jens Greve, Gerda Wurpts, Pär Hallberg, Anette Bygum, Christian Von Buchwald, Malgorzata Karawajczyk, Michael Steffens, Julia Stingl, Per Hoffmann, Stefanie Heilmann-Heimbach, Elisabeth Mangold, Kerstin U. Ludwig, Eva R. Rasmussen, Mia Wadelius, Bernhardt Sachs, Markus M. Nöthen, Andreas J. Forstner

**Affiliations:** ^1^ Institute of Human Genetics, University of Bonn, School of Medicine and University Hospital Bonn, Bonn, Germany; ^2^ Institute for Genomic Statistics and Bioinformatics, University Hospital Bonn, Bonn, Germany; ^3^ Centre for Human Genetics, University of Marburg, Marburg, Germany; ^4^ Department of Dermatology and Allergy, Comprehensive Allergy Center, Hannover Medical School, Hannover, Germany; ^5^ Department of Dermatology, SLK Hospital Heilbronn, Heilbronn, Germany; ^6^ Department of Dermatology and Venereology, Medical University Graz, Graz, Austria; ^7^ Department of Dermatology, Venereology and Allergology, St. Josef Hospital, University Medical Center, Ruhr University Bochum, Bochum, Germany; ^8^ Department of Dermatology and Allergy, University Hospital of Bonn, Bonn, Germany; ^9^ Department of Dermatology, Venereology and Allergology, University Medical Center Göttingen, Göttingen, Germany; ^10^ Department of Otorhinolaryngology—Head and Neck Surgery, Ulm University Medical Center, Ulm, Germany; ^11^ Department of Dermatology and Allergy, Aachen Comprehensive Allergy Center, University Hospital RWTH Aachen, Aachen, Germany; ^12^ Department of Medical Sciences, Clinical Pharmacology and Science for Life Laboratory, Uppsala University, Uppsala, Sweden; ^13^ Department of Clinical Genetics, Odense University Hospital, Odense, Denmark; ^14^ Clinical Institute, University of Southern Denmark, Odense, Denmark; ^15^ Department of Otorhinolaryngology—Head and Neck Surgery and Audiology, Rigshospitalet, University of Copenhagen, Copenhagen, Denmark; ^16^ Department of Medical Sciences, Clinical Chemistry, Uppsala University, Uppsala, Sweden; ^17^ Research Division, Federal Institute for Drugs and Medical Devices, Bonn, Germany; ^18^ Institute for Clinical Pharmacology, RWTH Aachen University, Aachen, Germany; ^19^ Institute of Neuroscience and Medicine (INM-1), Research Center Jülich, Jülich, Germany

**Keywords:** angiotensin-converting enzyme inhibitor, angiotensin receptor blocker, angioedema, sequencing, genetics, hereditary angioedema

## Abstract

Angioedema is a relatively rare but potentially life-threatening adverse reaction to angiotensin-converting enzyme inhibitors (ACEi) and angiotensin receptor blockers (ARBs). As with hereditary forms of angioedema (HAE), this adverse reaction is mediated by bradykinin. Research suggests that ACEi/ARB-induced angioedema has a multifactorial etiology. In addition, recent case reports suggest that some ACEi/ARB-induced angioedema patients may carry pathogenic HAE variants. The aim of the present study was to investigate the possible association between ACEi/ARB-induced angioedema and HAE genes *via* systematic molecular genetic screening in a large cohort of ACEi/ARB-induced angioedema cases. Targeted re-sequencing of five HAE-associated genes (*SERPING1*, *F12*, *PLG*, *ANGPT1*, and *KNG1*) was performed in 212 ACEi/ARB-induced angioedema patients recruited in Germany/Austria, Sweden, and Denmark, and in 352 controls from a German cohort. Among patients, none of the identified variants represented a known pathogenic variant for HAE. Moreover, no significant association with ACEi/ARB-induced angioedema was found for any of the identified common [minor allele frequency (MAF) >5%] or rare (MAF < 5%) variants. However, several non-significant trends suggestive of possible protective effects were observed. The lowest *p*-value for an individual variant was found in *PLG* (rs4252129, p.R523W, *p* = 0.057, *p*.adjust > 0.999, Fisher’s exact test). Variant p.R523W was found exclusively in controls and has previously been associated with decreased levels of plasminogen, a precursor of plasmin which is part of a pathway directly involved in bradykinin production. In addition, rare, potentially functional variants (MAF < 5%, Phred-scaled combined annotation dependent depletion score >10) showed a nominally significant enrichment in controls both: 1) across all five genes; and 2) in the *F12* gene alone. However, these results did not withstand correction for multiple testing. In conclusion, our results suggest that HAE-associated mutations are, at best, a rare cause of ACEi/ARB-induced angioedema. Furthermore, we were unable to identify a significant association between ACEi/ARB-induced angioedema and other variants in the investigated genes. Further studies with larger sample sizes are warranted to draw more definite conclusions concerning variants with limited effect sizes, including protective variants.

## 1 Introduction

Angioedema is a disorder characterized by self-limiting, localized, edematous swellings of subcutaneous, and submucosal tissues. In general, angioedema is classified into: 1) allergic angioedema, which is mediated by mast-cell mediators such as histamine and is commonly concomitant with urticaria; and 2) non-allergic angioedema, which is mediated by bradykinin (Maurer et al., 2022).

Also, within bradykinin-mediated angioedema, two main groups are recognized: 1) hereditary angioedema (HAE); and 2) acquired forms, e.g., drug-induced (Maurer et al., 2022). Most commonly affected by such angioedema are the skin, respiratory tract or even the gastrointestinal tract, and in the case of upper airway involvement the angioedema might even be a potentially life-threatening event for patients (Caballero et al., 2011).

In HAE, disease-causing mutations have been reported in the genes *SERPING1* (Ponard et al., 2020), *F12* (Cichon et al., 2006; Dewald and Bork, 2006), *ANGPT1* (Bafunno et al., 2018), *PLG* (Bork et al., 2018), and *KNG1* (Bork et al., 2019). In contrast, research suggests that the etiology of drug-induced angioedema, in particular that arising in response to treatment with angiotensin-converting enzyme inhibitors (ACEi), is multifactorial (Marcelino-Rodriguez et al., 2019).

In general, angioedema is a rare adverse drug reaction (ADR) of ACEi, occurring in only 0.1%–0.7% of treated patients (Miller et al., 2008; Banerji et al., 2017). Owing to widespread ACEi prescribing, however, estimates suggest that up to 35,000 patients are affected annually in Germany alone (Bas, 2017). In addition, angioedema has also been observed in patients who receive angiotensin receptor blockers (ARB), which, as ACEi, act on the renin-angiotensin system (Makani et al., 2012; Rasmussen et al., 2019).

Research suggests that a central mechanism in the development of angioedema during ACEi therapy is the impaired bradykinin degradation, with the resulting increase in bradykinin levels leading to blood vessel dilatation, an increase in the vascular permeability of endothelial cells, and ultimately the clinically observable tissue swelling (Kaplan, 2008; Cicardi et al., 2014). ARBs were found to increase bradykinin levels as well, however, the exact mechanisms are not yet fully understood (Campbell et al., 2005; Bas et al., 2007).

Recent genome-wide association studies (GWAS) have identified the first genome-wide significant variants for ACEi/ARB-induced angioedema. These include intronic variants in the *KCNMA1* gene as well as variants located in an upstream region of the *BDKRB2* gene (Rasmussen et al., 2020; Ghouse et al., 2021). Furthermore, a recent exome sequencing study revealed associations between ACEi/ARB-induced angioedema and the common factor V Leiden mutation, as well as other rare variants in the *F5* gene (Maroteau et al., 2020). Despite these advances, however, the precise underlying genetic causes of ACEi/ARB-induced angioedema remain largely unclear.

Moreover, recent case reports have described the identification of HAE mutations in the genes *F12* and *PLG* in patients who had initially been diagnosed with ACEi-induced angioedema (Veronez et al., 2017; Germenis et al., 2018; Yakushiji et al., 2018). This suggests that some patients diagnosed with ACEi/ARB-induced angioedema are actually HAE cases, who cannot be distinguished on the basis of either clinical symptoms or medical history (Firinu et al., 2019).

The aim of the present study was to 1) screen ACEi/ARB-induced angioedema cases for previously described pathogenic HAE-associated variants and 2) further explore the role of other variants identified in these genes in a case-control setting. For this purpose, systematic molecular genetic screening of five genes associated with HAE was conducted in a large ACEi/ARB-induced angioedema cohort.

## 2 Materials and Methods

### 2.1 Study Sample

The cohort comprised ACEi/ARB-induced angioedema patients from Germany/Austria, Sweden, and Denmark, and controls from a German cohort.

German/Austrian cases (*n* = 67, 49.3% female, 50.7% male) were recruited within the context of the vARIANCE (Angioedema Risk Under Angiotensin-Converting Enzyme Inhibitors, www.variance-studie.info) study, whose aim is to identify risk factors for ACEi/ARB-induced angioedema in patients. Patients for the study are recruited in multiple hospitals and primary care centers in Germany and one hospital in Austria. All patients in the vARIANCE study were clinically assessed by experienced specialists from the respective recruitment center. Considering all clinical information, the enrolling specialist further evaluated the causality between the intake of the ACEi/ARB and the angioedema that occurred. Inclusion criterion for the present study was the assignment of a certain, probable/likely, or possible causal relationship between the occurrence of angioedema and the intake of an ACEi or ARB by the reporting physician. Exclusion criterion was a self-reported history of concomitant urticaria.

Swedish cases (*n* = 77, 54.4% female, 45.6% male) were recruited by the Swedegene project (www.swedegene.se). This nationwide biobank contains the clinical data and blood samples of various ADR cases.

Danish cases (*n* = 68, 39.0% female, 61.0% male) were recruited from multiple Danish hospitals, as well as *via* a collaborating general practitioner.

For both the Swedish and Danish cases, the clinical diagnosis of an ACEi/ARB-induced angioedema was initially made by the reporting physician, while it was later reviewed and adjudicated by a clinical expert in allergology using published phenotype standardization criteria (Wadelius et al., 2014). Briefly, patients who developed a swelling in the head or neck area during ACEi/ARB treatment that was diagnosed as angioedema by a physician were considered cases. Exclusion criteria for the Swedish and Danish cases were: 1) concomitant urticaria; 2) other suspected causes for the angioedema; and 3) a history of HAE or acquired C1-INH deficiency. Detailed information on recruitment and phenotype characterization in the Swedish and Danish samples is provided elsewhere (Rasmussen et al., 2020).

Controls (*n* = 352, 56.8% female, 43.2% male) were randomly selected from a cohort of healthy, voluntary blood donors of German descent (Birnbaum et al., 2009). Controls were not screened for a history of ACEi/ARB intake or ACEi/ARB-induced angioedema.

All participants provided written informed consent prior to inclusion. The study was approved by the respective institutional ethics committees.

### 2.2 Removal of Relatives and Ancestral and Population Outliers

Related individuals were identified using kinship analysis. In addition, principal component analyses (PCA) were performed to identify ancestral and population outliers, to avoid potential population stratification biases. Since no exome-wide sequencing data were available, these analyses were based on common variants derived from genome-wide genotype data. Genotyping of cases was performed using the Illumina Global Screening Array (GSA) v2.0. Genotyping of controls was performed using GSA v3.0. In view of these differing GSA versions, the following analyses were carried out using the overlapping single nucleotide polymorphism (SNP) content only (total of *n* = 701,178 SNPs). Kinship was assessed up to 3^rd^ degree relatives (kinship coefficient >0.0442) using KING (Kinship-based Inference for GWAS) (Manichaikul et al., 2010). In the case of related pairs, one individual from each pair (controls were removed in favor of cases) was excluded from further analysis. For the subsequent PCA, SNPs were filtered according to the following criteria: 1) call rate > 0.98; 2) Hardy-Weinberg equilibrium test *p*-value < 1e-3; and 3) minor allele frequency (MAF) > 0.01. The SNPs were then pruned (--indep-pairwise 1000 50 0.2) using PLINK1.9 (Chang et al., 2015). Ancestry was inferred using the --pca --projection option of the KING software (Manichaikul et al., 2010), and the 1000 Genomes (Auton et al., 2015) as a reference dataset. Samples with assigned Non-European ancestry were removed as ancestral outliers. To account for potential population outliers within the study cohort, a subsequent PCA was performed using the --pca option of PLINK1.9 (Chang et al., 2015). Samples deviating >6 standard deviations from the mean in any of the first 10 PCs were defined as outliers.

### 2.3 Selection of Candidate Genes

Genes carrying previously described pathogenic HAE mutations were identified using the Human Gene Mutation Database [HGMD professional, query mid-2019; (Stenson et al., 2020)]. In addition, a literature search was performed to identify genes not listed in the HGMD, but reported to carry pathogenic variants for HAE. Using these two strategies, we identified five genes which were included in the present study: *SERPING1*, *F12*, *PLG*, *ANGPT1*, and *KNG1*. Supplementary Table S1 summarizes the previous studies that implicated the five HAE-associated genes.

### 2.4 Targeted Candidate Gene Sequencing

#### 2.4.1 Design of Single-Molecule Molecular Inversion Probes

Sequencing of the five candidate genes was performed using 116 single-molecule molecular inversion probes (smMIPs, 10.1 kb target region) that had been designed as a part of a larger candidate gene panel comprising a total of 29 genes (718 smMIPs, 69.4 kb target region).

The smMIP design process was carried out using an in-house pipeline (Thieme et al., 2021) based on the MIPgen software (Boyle et al., 2014), and was targeted to cover all candidate gene exons and exon/intron boundaries (±6 bp flanking sequence), as based on RefSeq gene definitions (O’Leary et al., 2016). For the gene *F12*, which was associated in previous research with an estrogen-dependent HAE subtype (Binkley, 2010), an estrogen-response element located in the 5’ UTR (Farsetti et al., 1995) was also included in the primer design. For variants with a MAF >1% [in the Non-Finnish European population in the gnomAD database v.2.1.1, (Karczewski et al., 2020)] occurring in either the extension arm or the ligation arm of smMIPs, ambiguous coding was used for the respective base in order to obtain a primer mix with 50% reference and 50% alternative allele. Primer re-design was performed for smMIPs with logistic scores <0.6, and/or a product of extension and ligation arm copy >20, and/or insertion/deletion variants within extension or ligation arms. Additionally, proper exon coverage of the final smMIP design was visually verified using the UCSC Genome Browser (Kent et al., 2002). Selected oligonucleotides (100 µM) were ordered from Integrated DNA Technologies (IDT, Leuven, Belgium). The sequences of the 116 smMIPs used in this study are listed in the Supplementary Table S2.

#### 2.4.2 Library Preparation

DNA concentration was quantified using a Quant-iT PicoGreen dsDNA Assay (Thermo Fisher Scientific, Waltham, MA, United States), and diluted to 4 ng/μl. Input for the final reaction was 50 ng. All 116 smMIPs were pooled in equimolar ratios and phosphorylated, as described elsewhere (Eijkelenboom et al., 2016; Thieme et al., 2021), with minor modifications, and an smMIP to DNA ratio of 1,600:1. To adjust for over- and under-performing smMIPs, two (re-)balancing runs were performed using six and two test samples, respectively. (Re-)balancing was carried out for all 718 smMIPs of the 29 candidate gene panel using an Illumina MiSeq (v2 nano kit). The results were then used to adjust the 116 smMIPs included in the present study.

Library preparation, which included phosphorylation of smMIPs, hybridization, exonuclease treatment, and polymerase-chain-reaction (PCR), was performed as described elsewhere (Eijkelenboom et al., 2016; Thieme et al., 2021) with the following modification: PCR for the second sequencing run was performed using two sample-specific barcoded primers (1.25 µl respectively, 10 µM, IDT, Coralville, IA, United States), while for the first sequencing run, only one primer contained a sample-specific barcode.

PCR products were pooled in a plate-wise manner, and purified using 0.8x volume of Agencourt AMPure XP beads (Beckman-Coulter, Brea, CA, United States). The purity and fragment length of the individual pools were assessed on an Agilent 2200 TapeStation system using HSD1000 ScreenTapes, and the concentration was measured using a Qubit dsDNA HS Assay (Thermo Fisher Scientific, Waltham, MA, United States).

#### 2.4.3 Single-Molecule Molecular Inversion Probes Sequencing

For re-sequencing, the individual pools were pooled equimolarly into two mega-pools (final concentration 3 nM). These contained 111 samples (cases only) and 440 samples (92 cases, 348 controls), respectively. For both mega-pools, sequencing was performed on an Illumina NextSeq550 platform using 2 × 150 bp paired-end sequencing in mid-output mode and custom sequencing primers [IDT, Leuven, Belgium; (O’Roak et al., 2012)], in accordance with the Illumina protocol.

For privacy reasons, the sequence data of the current study cannot be uploaded to an online repository. The data are available from the authors upon request.

### 2.5 Data Analysis

Demultiplexing of the base call files and conversion to FASTQ files was performed using the bcl2fastq conversion software (Illumina, San Diego, CA, United States). Bioinformatic analysis of the FASTQ files was conducted using an in-house analysis pipeline that is based on steps as described elsewhere (Hiatt et al., 2013). Briefly, paired-end reads were merged using PEAR [Paired-End reAd mergeR, (Zhang et al., 2014)], and aligned to the hg19 reference genome using BWA-MEM (Li and Durbin, 2009). Using available MIPgen scripts (Boyle et al., 2014), smMIPs were trimmed and collapsed. This step involved the use of molecular tags (introduced as five degenerated bases during synthesis of the smMIPs) to collapse reads carrying the same tag into single reads, thereby creating high consensus sequences and reducing PCR artifacts. Variant calling was performed using GATK UnifiedGenotyper (van der Auwera et al., 2013), and the retrieved variants were annotated using ANNOVAR (Wang et al., 2010). Homozygous and heterozygous call thresholds were set to alternative alleles occurring in >75% or >25% of reads, respectively.

### 2.6 Quality Control

For quality control (QC), BAM files derived from the smMIP analysis pipeline were used to determine a bp-wise coverage of the whole target region using samtools 0.1.19 (Li et al., 2009), R (R Core Team, 2020), and the R packages tidyverse (Wickham et al., 2019), ggplot2 (Wickham, 2016), data.table (Dowle and Srinivasan, 2021), and splitstackshape (Mahto, 2019).

To answer the two different research questions that this study seeks to address, data QC was performed in two stages and separately in cases and controls. Following QC, the identified variants were merged for subsequent analyses.

In stage one, samples that did not achieve a mean coverage of 50× and/or a total coverage of more than 15× in at least 90% of the target region were excluded. To ensure proper coverage of known disease-causing mutations, the mean coverage of all such variants, as listed in the professional HGMD [version 04.2020, (Stenson et al., 2020)], was examined separately for first run and second run cases as well as for controls. Exons with known mutations in regions with a coverage of <20× in either one of the three groups were excluded from further analysis and re-assessed using Sanger sequencing. In addition, low-quality variants (QUAL < 30 and QD < 10) were excluded. In stage two, to only obtain high-confidence variants, further filtering was performed by excluding variants that did not have a coverage of <15× in at least 90% of the sequenced samples.

To ensure comparable coverage between the two sequencing runs and cases and controls, a group-wise comparison of the mean coverages was performed using a Kruskal–Wallis rank sum test [kruskal.test() function of the R stats package (R Core Team, 2020)].

### 2.7 Sanger Sequencing

Sanger sequencing was performed to investigate two exons (exon 9 of *F12* and exon 3 of *SERPING1*) with known HAE disease-causing mutations in low coverage regions (<20× in smMIP sequencing). In total, 195 of 197 cases and all 346 controls (numbers after sample QC) were sequenced. Two Danish cases could not be re-sequenced due to a lack of DNA material. Sequencing was performed using standard PCR conditions and the Sanger sequencing service of GENEWIZ (GENEWIZ Europe by Azenta Life Science, Leipzig, Germany). Primer sequences are listed in Supplementary Table S3. Generated sequences were analyzed using the SeqMan II software (DNASTAR, Madison, WI, United States). Variants identified in the sequences were aligned to the human reference genome (hg19) using the BLAT tool of the UCSC Genome Browser (Kent et al., 2002) and then jointly analyzed with the variants obtained *via* smMIP sequencing.

### 2.8 Evaluation of the Sequencing Data for Known Causative Hereditary Angioedema Mutations

All variants identified in cases after QC stage one were individually queried in the professional version of the HGMD [version 04.2020, (Stenson et al., 2020)] in order to determine whether they were a reported pathogenic mutation for HAE.

### 2.9 Variant Prioritization and Statistical Analysis

Variants remaining after QC stage two were further prioritized for rare, potentially functional variants using the following criteria: 1) MAF < 0.05 (based on the combined cases and controls of the present study); and 2) PHRED-scaled CADD score >10 [combined annotation dependent depletion score; version 1.6 (Rentzsch et al., 2021)]. To test the identified variants for significant case-control differences, a Fisher’s exact test was performed using the fisher.test() function of the R stats package (R Core Team, 2020). Additionally, a Fisher’s exact test was calculated to test if the number of samples carrying at least one prioritized variant differed significantly between cases and controls. This was tested: 1) gene-wise; and 2) across all five genes. *p*-values < 0.05 were considered nominally significant. To correct for multiple testing, adjusted *p*-values were calculated using the Bonferroni method taking into account the number of individual variants (*n* = 85) and the number of investigated genes plus the entire gene panel (*n* = 5 + 1 = 6).

### 2.10 Power Calculation

We used the R package genpwr (Moore et al., 2020) to determine the power in our final analysis sample (N_total_ = 543, case rate = 0.36) to detect rare variants (MAF <0.05 or <0.01) within a range of different effect sizes. Using an additive genetic model and a significance level of 0.05, we had 80% power to detect an odds ratio (OR) of 2.2 for variants with a MAF <0.05. For rarer variants with MAF <0.01, we had a power of 80% to detect an OR of 6.2 (Supplementary Figure S1a). We further calculated the number of samples that would have been required to detect variants with a MAF <0.05 and lower ORs (additive genetic model, 80% power, significance level 0.05). With an approximately 2-fold increase in sample size compared to the present study (N_total_ = 1,082, case rate = 0.36) we would have been able to detect variants with an OR of around 1.75 (Supplementary Figure S1b).

## 3 Results

### 3.1 Pre-Sequencing Sample Quality Control

Kinship analysis revealed five pairs of first degree relatives within the study sample (one pair of cases, four pairs of controls). Using 1000 Genomes as a reference data set, four cases with non-European ancestry were identified (Supplementary Figure S2a). A further four samples were found to be population outliers in the subsequent PCA of the study sample (Supplementary Figure S2b). After the removal of relatives and ancestral and population outliers, a total of 203 cases and 348 controls underwent sequencing.

### 3.2 Identification and Annotation of Variants

The two sequencing runs showed mean coverages of 245-fold and 239-fold, respectively. The group-wise comparison of the mean coverages revealed no significant inter-run differences between cases and controls. However, cases sequenced in the second run showed a significantly lower coverage than that observed for cases sequenced in the first run and controls sequenced in the second run (Supplementary Figure S3). Despite this, and given that the mean coverage in all three groups was still well above 200-fold, this should not have had a major impact on variant detection. Sample-level QC criteria were fulfilled by 543 samples (>98%, 6 cases and 2 controls were excluded). Overall, 74 and 104 variants were identified in cases and controls, respectively. Among cases, 18 variants were excluded in variant-level QC stage 1, while six additional variants were excluded in stage 2. Variant-level QC in controls led to the exclusion of a total number of 31 variants.

### 3.3 Baseline Characteristics of the Final Analysis Sample

The final analysis included 197 ACEi/ARB-induced angioedema cases (46.5% females, 53.5% males) and 346 controls (55.8% females, 44.2% males). The mean age at event (development of angioedema) in cases was 65.1 years, and for the majority of patients (71.3%) more than 1 year elapsed between the first intake of an ACEi/ARB and the onset of angioedema. The suspected drug was reported to be an ACEi in 87% of all cases, and an ARB in the remaining 13%. However, the most commonly suspected drug varied between the case cohorts, being: 1) Ramipril in the German cases (66% of all ACEi cases); and 2) Enalapril in the Danish and Swedish cases (67.3% and 92% of all ACEi cases, respectively). More detailed information on the baseline characteristics of the sample included in the final analysis is provided in Table 1.

**TABLE 1:** Baseline characteristics of the final study sample.

		Cases			Controls (*n* = 346)
		German (*n* = 64)	Danish (*n* = 62)	Swedish (*n* = 71)	
Female:male (%)		50.0:50.0	51.6:48.4	38.0:62.0	55.8:44.2
Mean age at event (years, range)		63.2 (43–85)	65.1 (37–89)	67.0 (42–86)	NA
Time to onset, N (%)	1-3d	2 (3.2%)	3 (9.1%)	4 (6.5%)	NA
	4-14d	4 (6.5%)	0	3 (4.8%)	
	2w-2m	2 (3.2%)	0	3 (4.8%)	
	2m-1y	5 (8.1%)	3 (9.1%)	16 (25.8%)	
	>1y	49 (79.0%)	27 (81.8%)	36 (58.1%)	
Suspected ACEi, N (%)		50 (82.0%) of that: Ramipril, 33 (66.0%); Lisinopril, 10 (20.0%); Enalapril, 7 (14.0%)	55 (90.2%) of that: Enalapril, 37 (67.3%); Ramipril, 8 (14.5%); Trandolapril, 4 (7.3%); Lisinopril, 3 (5.5%); Perindopril, 1 (1.8%); NA, 1 (1.8%)	63 (88.7%) of that: Enalapril, 58 (92.0%); Ramipril, 5 (7.9%)	NA
Suspected ARB, N (%)		11 (18.0%) of that: Candesartan, 5 (45.5%); Valsartan, 3 (27.3%); Telmisartan, 2 (18.2%); Irbesartan, 1 (9.1%)	6 (9.8%) of that: Losartan, 5 (83.3%); Irbesartan, 1 (16.7%)	8 (11.3%) of that: Candesartan, 5 (62.5%); Losartan, 3 (37.5%)	NA

For one German patient and two Danish patients, age at event was not available. For two German, 29 Danish, and nine Swedish cases, no data on the time interval between the first intake and the occurrence of angioedema (“time-to-onset”) was available, corresponding percentages were therefore calculated on the basis of *n* = 62, *n* = 33, and *n* = 62 for the German, Danish, and Swedish patients, respectively. Data on suspected ACEi/ARB drug are based on cases for which complete information was available. For one Danish case, no information on suspected drug was available. For three German cases, two different suspected drugs were documented. Thus, for the Danish and German cases, the respective percentages were calculated on the basis of *n* = 61 samples. Abbreviations: ACEi, angiotensin-converting enzyme inhibitor; ARB, angiotensin receptor blocker; d, day(s); w, weeks; m, months; y, year; NA, not available.

### 3.4 Evaluation of Known Hereditary Angioedema Mutations in ACEi/ARB-Induced Angioedema Cases

After the exclusion of low-quality variants (QUAL < 30, QD < 10), a total of 56 variants were identified in cases. Screening of these variants in the professional version of HGMD revealed that none represented a known pathogenic HAE variant. As an incidental finding, the HGMD query yielded a missense variant in *F12* (rs35515200; NM_000505.4:c.418C>G; p.L140V), which is associated with deep vein thrombosis according to Lotta et al. (2012). In the present cohort, this was found in a heterozygous state in two patients and three controls. However, re-evaluation of the variant using the ACMG guidelines for the interpretation of sequence variants (Richards et al., 2015) revealed that this is a variant of uncertain significance.

### 3.5 Identified and Prioritized Variants, and Association Results

After the exclusion of low-confidence variants (<15× in ≥10% of samples), a total of 85 independent variants were identified. These included 15 common (MAF > 0.05) and 70 rare variants (MAF < 0.05). Further prioritization resulted in 42 rare, potentially functional variants with a CADD score >10. All rare variants were found in a heterozygous state, with the exception of two variants, i.e., one in *F12* and one in *PLG*, which were identified in a homozygous state in one case and one control, respectively.

A single variant level Fisher’s Exact test showed that none of the identified common or rare variants were significantly associated with ACEi/ARB-induced angioedema. The top five variants, i.e., those with the lowest *p*-values, are listed in Table 2.

**TABLE 2:** Top five rare candidate variants identified *via* re-sequencing.

Gene	Genomic location (hg19)	Ref/Alt	AA change	rsID (dbSNP155)	Consequence	HGVS nomenclature	CADD v1.6	AC case/ctrl	*p-*value	*p*.adjust
*PLG*	6:161152905	C/T	R523W	rs4252129	Nonsynonymous SNV	NM_000301.5:c.1567C>T	19.54	0/8	0.057	>0.999
*F12*	5:176831826	C/G	A207P	rs17876030	Nonsynonymous SNV	NM_000505.4:c.619G>C	13.18	2/14	0.064	>0.999
*KNG1*	3:186461524	C/T	R376X, R412X	rs76438938	Stopgain	NM_001166451.2:c.1126C>T, NM_000893.4:c.1234C>T	16.28	6/24	0.081	>0.999
*PLG*	6:161134069	G/A	R153R	rs144153702	Synonymous SNV	NM_000301.5:c.459G>A	11.19	2/0	0.131	>0.999
*PLG*	6:161137790	G/A	R261H	rs4252187	Nonsynonymous SNV	NM_000301.5:c.782G>A	27.70	4/3	0.263	>0.999

Abbreviations: hg19, genomic location according to the Genome Reference Consortium human build 37 (GRCh37); Ref, reference allele; Alt, alternative allele; AA, amino acid; rsID, reference SNP cluster ID; SNV, single nucleotide variant; HGVS, human genome variant society; CADD v1.6, Phred-scaled combined annotation dependent depletion score version 1.6; AC case/ctrl, allele counts in cases and controls (sample = 197 cases, 346 controls); *p*.adjust, Bonferroni adjusted *p*-value.

Across all genes, the number of individuals who carried at least one prioritized variant (MAF < 0.05 and CADD > 10) was statistically higher among controls than among cases (*p* = 0.015). However, this result did not withstand correction for multiple testing (*p*.adjust = 0.091). In general, the same trend was observed at the gene-level. Overall, more controls than cases carried at least one prioritized variant, as shown in Table 3. The difference, though, was only significant for *F12* (*p* = 0.033) and this nominally significant finding did not withstand correction for multiple testing (*p*.adjust = 0.199).

**TABLE 3:** Enrichment analysis of rare, potentially functional variants in the five investigated genes.

Gene	Cases carrying ≥1 prioritized variant		Controls carrying ≥1 prioritized variant		*p*-value	*p*.adjust
	N	%	N	%		
*ANGPT1*	8	4.1	22	6.4	0.330	>0.999
*F12*	4	2.0	21	6.1	**0.033**	0.199
*KNG1*	21	10.7	57	16.5	0.075	0.448
*PLG*	19	9.6	37	10.7	0.770	>0.999
*SERPING1*	1	0.5	5	1.4	0.425	>0.999
Whole panel	47	23.9	118	34.1	**0.015**	0.091

Abbreviations: N, number of individuals; *p*.adjust, Bonferroni adjusted *p*-value.

Bold font indicates nominally significant *p*-values.

## 4 Discussion

In the present study, systematic molecular genetic screening was performed to investigate the presence of known disease-causing HAE mutations in a large cohort (*n* = 197) of patients with ACEi/ARB-induced angioedema. This involved the targeted sequencing of five genes (*SERPING1*, *F12*, *PLG*, *ANGPT1*, and *KNG1*) with a previously reported association to HAE. In addition, analyses were performed to determine the contribution of additional variants in these genes to ACEi/ARB-induced angioedema.

None of the present ACEi/ARB-induced angioedema patients carried a pathogenic variant that is associated with HAE according to HGMD. This is consistent with the results of a smaller, previous study by Carucci et al. (2020), which found no disease-causing mutations in *SERPING1*, *F12*, *PLG,* or *ANGPT1* in 33 patients with ACEi-induced angioedema. Together, these results and the findings of the present study suggest that HAE causing mutations are, at best, a rare cause of ACEi/ARB-induced angioedema.

Association analysis at the individual variant level revealed no statistically significant differences in identified variants between cases and controls, neither for common nor for rare variants. This appears to be consistent with the results of the exome study of Maroteau et al. (2020), which did not report any of the presently investigated genes in their list of the most strongly associated genes. However, given the limited sample size of the present study, the non-significant results could also be attributable to a lack of statistical power. Although, our study had 80% power to detect rare variants (MAF <0.05) with an OR of 2.2, we were lacking the power to detect variants with small to moderate effect sizes. As shown in the sample size calculation we performed, the identification of such variants would require larger sample sizes to detect statistically significant differences between cases and controls.

A nominally significant enrichment of prioritized variants (MAF < 0.05, CADD > 10) was found in controls 1) across all five genes combined and 2) on a single gene-level for the *F12* gene, possibly pointing to the existence of variants with protective effects. However, these associations did not withstand correction for multiple testing.

The variant with the lowest *p*-value, thus representing the most promising finding, was a missense variant (rs4252129; NM_000301.5:c.1567C>T; p.R523W) in the kringle 5 domain of the gene *PLG*. This variant was only identified in controls. A previous study found that the minor allele of this variant was associated with decreased plasminogen levels (average decrease of around 14%) (Ma et al., 2014). In the context of ACEi/ARB-induced angioedema, a plausible hypothesis is that decreased plasma plasminogen levels lead to naturally lower bradykinin levels, in which case this variant may be protective against bradykinin accumulation during treatment with ACEi/ARB. With regard to this finding, the present study was limited by the use of unscreened controls. Further investigation is required to confirm whether this variant is also present in patients who are taking an ACEi/ARB but do not develop angioedema.

The top findings also included the stop variant p.R412X, which is located in exon 11 of the gene *KNG1* (rs76438938; NM_00893.4:c.1234C>T; p.R412X). Kininogen is the precursor for high molecular weight kininogen (HK) and low molecular weight kininogen (LK), which are produced *via* alternative splicing of *KNG1* (Colman and Schmaier, 1997). The HAE associated *KNG1* mutation is located in exon 10 of the gene, and directly affects the cleavage-site of bradykinin (Bork et al., 2019). The stop variant identified in the present study is located in exon 11, and is thus located downstream of both the bradykinin sequence and its cleavage sites. Exon 11 of *KNG1* encodes the unique light chain of LK, whose exact function has yet to be determined (Colman and Schmaier, 1997). The consequences of the identified stop variant p.R412X are therefore unclear, and functional studies would be required to elucidate its possible impact on ACEi/ARB-induced angioedema.

The present study had several limitations. First, despite representing one of the largest systematic investigations of the contribution of variants in the HAE genes to ACEi/ARB-induced angioedema to date, the study was underpowered in terms of the detection of statistically significant associations for variants with small to moderate effects. Second, since a control cohort was used that was not screened for a history of ACEi/ARB intake or ACEi/ARB-induced angioedema, the possibility that individuals from the control cohort would also be susceptible to angioedema during treatment with an ACEi or ARB cannot be excluded. However, given that the incidence for ACEi/ARB-induced angioedema is <1% (Makani et al., 2012; Bas, 2017; Rasmussen et al., 2019), the use of unscreened controls is unlikely to have had a major impact on the overall power of the present analyses (Moskvina et al., 2005). Third, in view of the lack of exome-sequencing data, PCA was performed on the basis of common variants derived from SNP-array data in order to account for potential population stratification. However, given that common and rare variants may differ in terms of stratification patterns (Mathieson and McVean, 2012), our approach may not have fully addressed differences in population substructure resulting from the different ancestries of the investigated cases and controls. Fourth, primers were designed to cover only the exonic regions ±6 bp flanking regions of the five investigated genes. Thus, we were unable to detect intronic or other regulatory variants in regions not covered by the selected primers, which have previously been reported for the *SERPING1* gene in a few HAE cases (Hujová et al., 2020; Ponard et al., 2020; Vatsiou et al., 2020). Fifth, the five here investigated genes do not explain the entire HAE phenotype, nor do they cover genes that may be involved in the phenotypic variation observed in HAE. In some patients, no genetic cause has yet been identified, and during the course of the present investigation, mutations in *MYOF* (Ariano et al., 2020), and *HS3ST6* (Bork et al., 2021), respectively, were identified and linked to two new HAE subtypes. Moreover, first studies have shown that variants in bradykinin-related genes (e.g., *KLKB1*) influence some of the phenotypic variations observed in HAE (Gianni et al., 2017), and it cannot be excluded that such variants may also play a role in the occurrence of ACEi/ARB-induced angioedema. Therefore, future studies are warranted to investigate the contribution of rare variants in novel HAE and HAE candidate genes to the development of ACEi/ARB-induced angioedema.

Further, larger studies of the whole exome or genome are warranted to assess the precise contribution of rare variants to ACEi/ARB-induced angioedema, particularly in genes implicated by recent GWAS (Rasmussen et al., 2020; Ghouse et al., 2021), which eventually could help to identify individuals with an increased risk to develop such angioedema. Ideally, these studies would use screened and treatment-matched controls in order to generate further insights into the potential role of protective variants. Beyond that, future studies may also examine the contribution of genetic variants to disease subphenotypes such as angioedema severity.

In conclusion, the present analyses identified no known disease-causing HAE variant in *SERPING1*, *F12*, *PLG*, *ANGPT1,* or *KNG1* in a cohort of around 200 patients with ACEi/ARB-induced angioedema. Moreover, no significant association was found between ACEi/ARB-induced angioedema and any other additional variants in these genes. However, the analyses identified a missense variant in the gene *PLG*, which might represent an interesting candidate for protection against ACEi/ARB-induced angioedema, since it has previously been associated with lower plasma levels of plasminogen (Ma et al., 2014), a protein which is involved in the formation of bradykinin (de Maat et al., 2018).

## Data Availability

All relevant data are within the paper and its Supplementary Material. The generated sequencing data are available from the authors upon request.
