# Supplementary material for: Molecular Genetic Screening in Patients With ACE Inhibitor/Angiotensin Receptor Blocker-Induced Angioedema to Explore the Role of Hereditary Angioedema Genes
Source: Front Genet. 2022 Jul 18;13:914376. doi: 10.3389/fgene.2022.914376 (PMC9339951; doi:10.3389/fgene.2022.914376)
Supplement: Supplementary file 1 [file DataSheet1.docx]

Molecular genetic screening in patients with ACE inhibitor/ angiotensin receptor blocker-induced angioedema to explore the role of hereditary angioedema genes

**Carina M. Mathey**^1^**, Carlo Maj**^2,3^**, Annika B. Scheer**^1^**, Julia Fazaal**^1^**, Bettina Wedi**^4^, **Dorothea Wieczorek^4^**, **Philipp M. Amann**^5^, **Harald Löffler**^5^, **Lukas Koch**^6^, **Clemens Schöffl**^6^, **Heinrich Dickel**^7^, **Nomun Ganjuur**^7^, **Thorsten Hornung**^8^, **Susann Forkel**^9^, **Jens Greve**^10^, **Gerda Wurpts**^11^, **Pär Hallberg**^12^, **Anette Bygum**^13,14^, **Christian von Buchwald**^15^, **Malgorzata Karawajczyk**^16^, **Michael Steffens**^17^, **Julia Stingl**^18^, **Per** **Hoffmann**^1^**, Stefanie Heilmann-Heimbach**^1^**, Elisabeth Mangold^1^, Kerstin U. Ludwig**^1^**, Eva R. Rasmussen**^15^**, Mia Wadelius**^12^**, Bernhardt Sachs**^11,17^**^†^, Markus M. Nöthen**^1^**^†^, Andreas J. Forstner**^1,19^**^†^***

**^†^**These authors have contributed equally to this work and share senior authorship.

^1^Institute of Human Genetics, University of Bonn, School of Medicine & University Hospital Bonn, Bonn, Germany**,** ^2^Institute for Genomic Statistics and Bioinformatics, University Hospital Bonn, Bonn, Germany**,** ^3^Centre for Human Genetics, University of Marburg, Marburg, Germany**,** ^4^Department of Dermatology and Allergy, Comprehensive Allergy Center, Hannover Medical School, Hannover, Germany**,** ^5^Department of Dermatology, SLK Hospital Heilbronn, Heilbronn, Germany**,** ^6^Department of Dermatology and Venereology, Medical University Graz, Graz, Austria**,** ^7^Department of Dermatology, Venereology and Allergology, St. Josef Hospital, University Medical Center, Ruhr University Bochum, Bochum, Germany**,** ^8^Department of Dermatology and Allergy, University Hospital of Bonn, Bonn, Germany**,** ^9^Department of Dermatology, Venereology and Allergology, University Medical Center Göttingen, Göttingen, Germany**,** ^10^Department of Otorhinolaryngology, Head and Neck Surgery, Ulm University Medical Center, Ulm, Germany**,** ^11^Department of Dermatology and Allergy, Aachen Comprehensive Allergy Center, University Hospital RWTH Aachen, Aachen, Germany**,** ^12^Department of Medical Sciences, Clinical Pharmacology and Science for Life Laboratory, Uppsala University, Uppsala, Sweden **,** ^13^Department of Clinical Genetics, Odense University Hospital, Odense, Denmark**,** ^14^Clinical Institute, University of Southern Denmark, Odense, Denmark**,** ^15^Department of Otorhinolaryngology – Head and Neck Surgery and Audiology, Rigshospitalet, University of Copenhagen, Copenhagen, Denmark **,** ^16^Department of Medical Sciences, Clinical Chemistry, Uppsala University, Uppsala, Sweden**,** ^17^Federal Institute for Drugs and Medical Devices, Research Division, Bonn, Germany**,** ^18^Institute for Clinical Pharmacology, RWTH Aachen University, Aachen, Germany**,** ^19^Institute of Neuroscience and Medicine (INM-1), Research Center Jülich, Jülich, Germany

Supplementary Figures


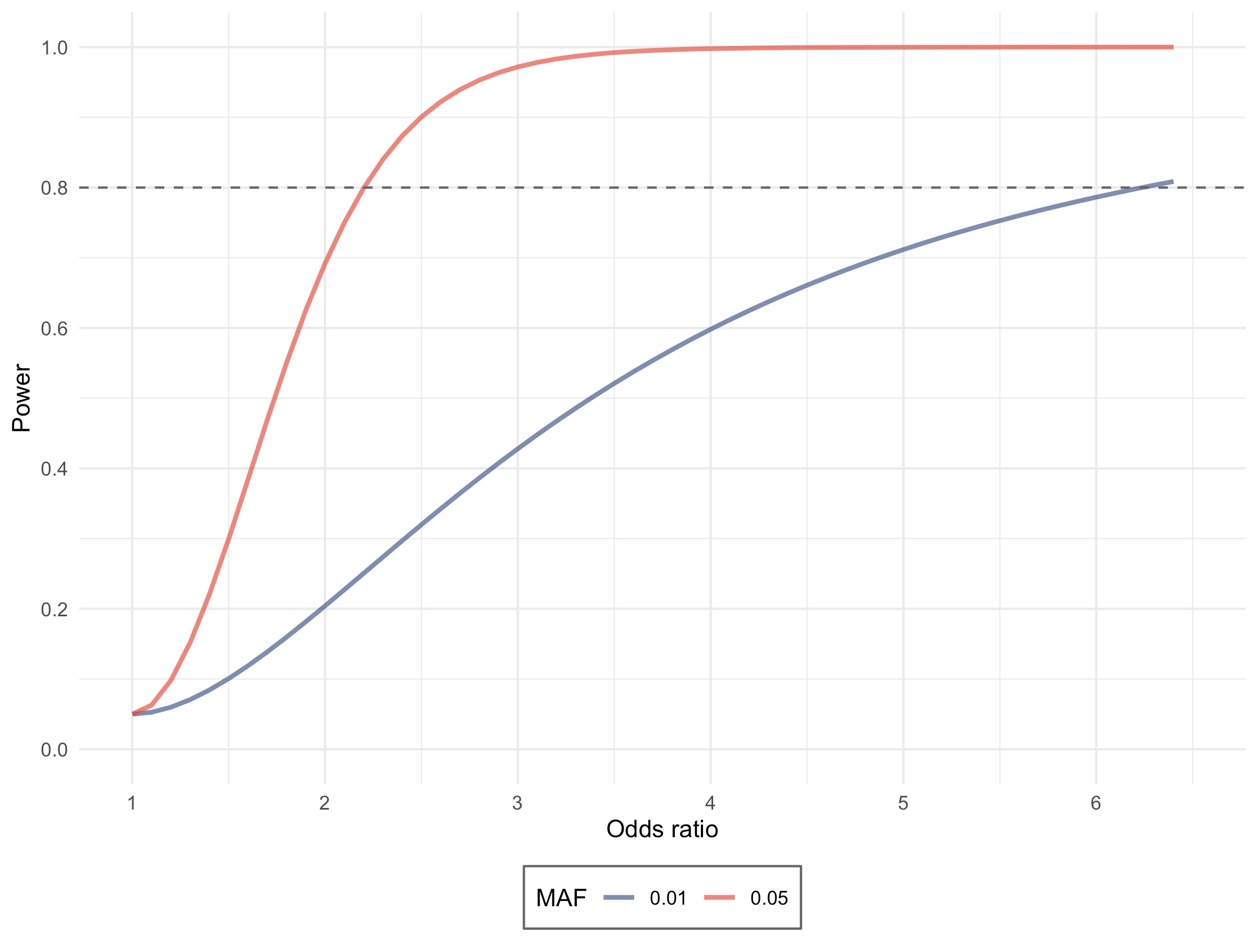


**Figure S1a | Power calculation.**

The power calculation was based on the final analysis sample (N_case_ = 197, N_ctrl_ = 346), a significance level of 0.05 and an additive genetic model. We had a power of 80% to detect variants with a MAF < 0.05 and an odds ratio (OR) of 2.2 as well as variants with a MAF < 0.01 and an OR of 6.2.

Abbreviations: MAF, minor allele frequency.


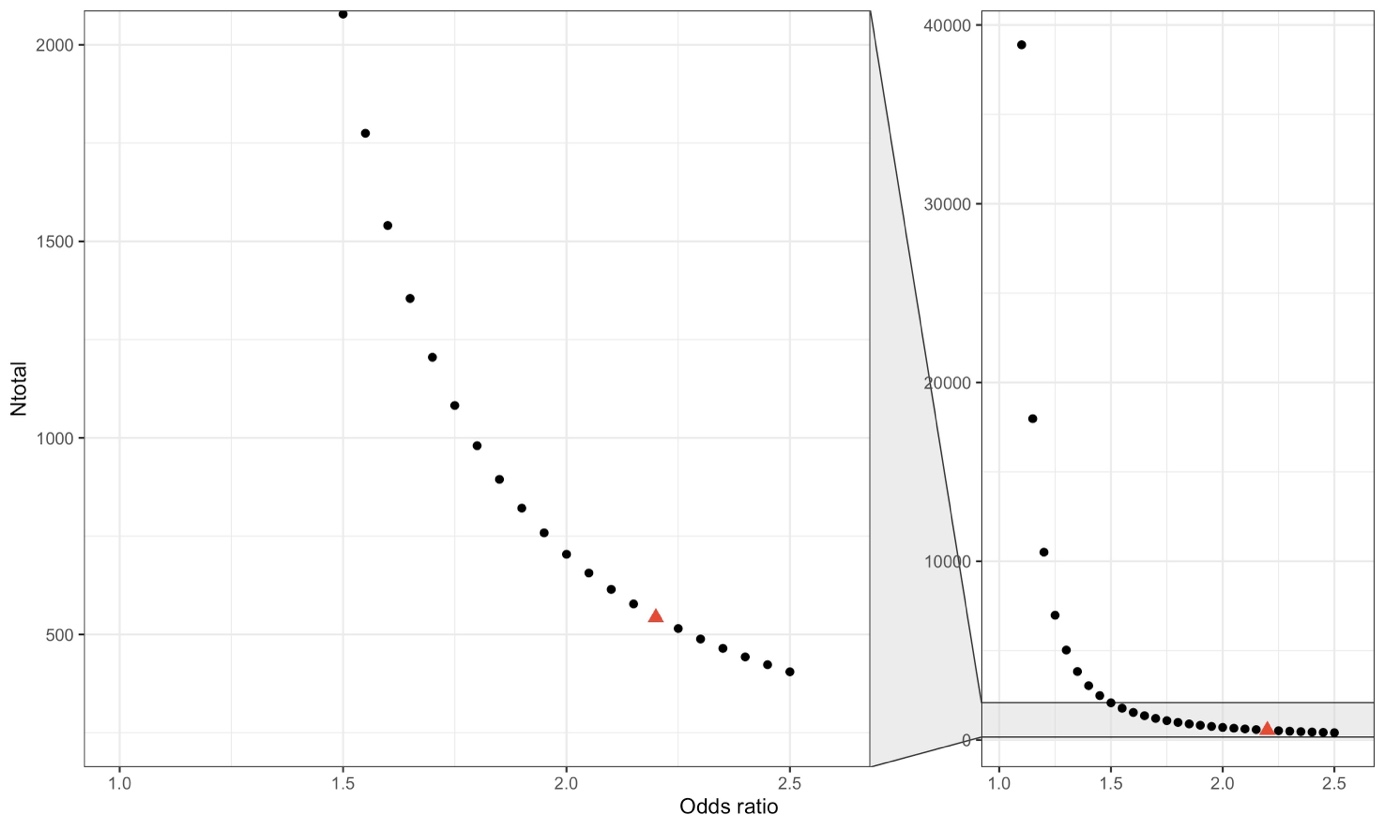


**Figure S1b | Sample size calculation.**

Figure S1b displays the total number of samples (N_total_) required to detect variants with lower odds ratios (ORs) compared with the sample size of the present study cohort, which is represented by the red triangle. The model is based on a case rate of 0.36, variants with a MAF < 0.05 and assumes an additive genetic model, a significance level of 0.05 and 80% power. For example, an approximately 2-fold increase in sample size (N_total_ = 1,082) would be required to detect variants with an OR of around 1.75.

*
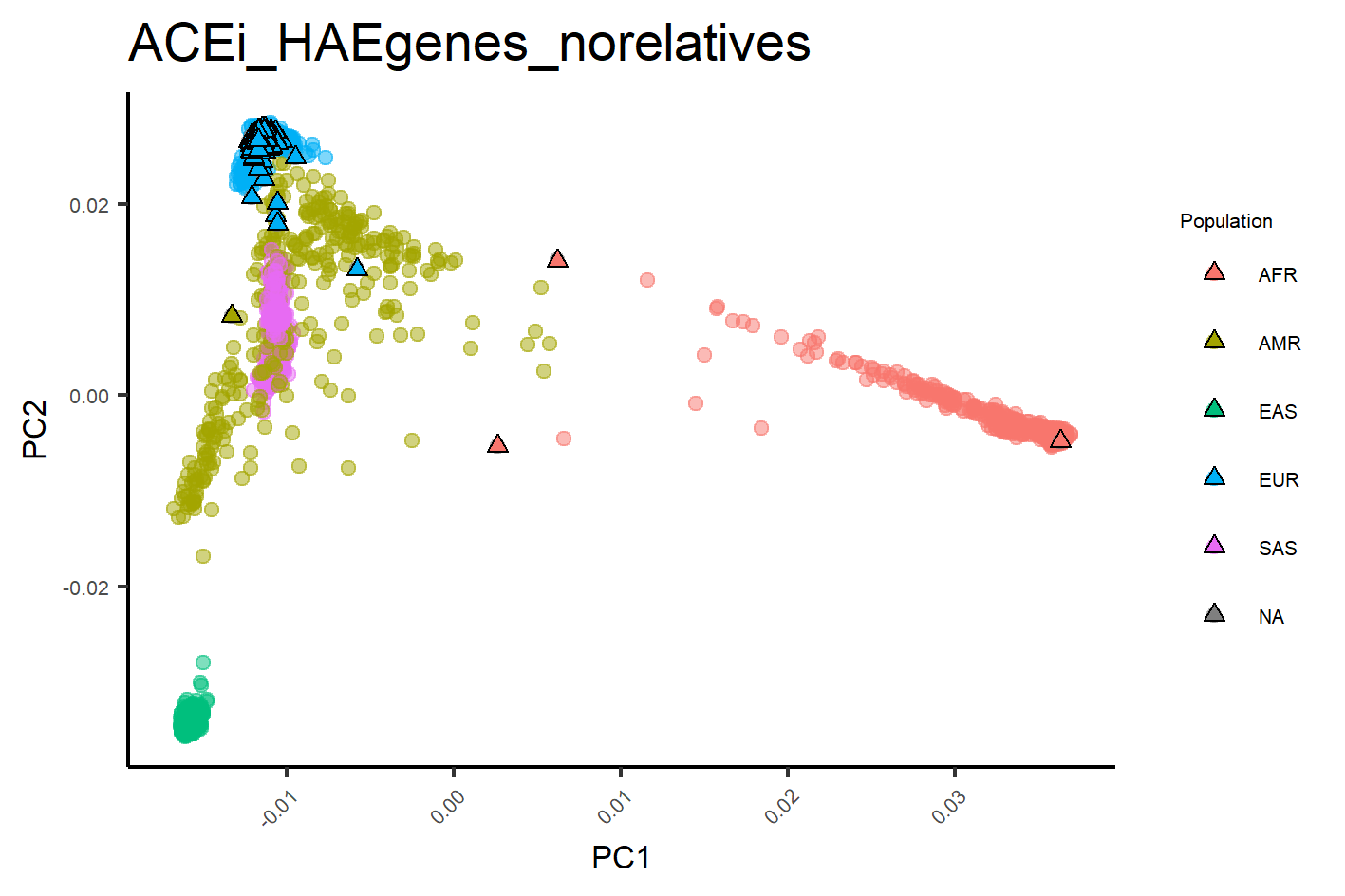
*

**Figure S2a | Plot of the first two genetic principal components for the study population and the 1000 Genomes reference dataset.**

Individuals of the 1000 Genomes dataset are represented by circles, colored according to their super population group. Individuals of the study sample are represented by black triangles, while the fill color of the triangles represents the inferred ancestry of the individuals. The vast majority of the study sample is located in the European cluster (blue), only four samples are outliers according to their inferred ancestry: three samples showed an African ancestry (red) and one sample showed an Ad Mixed American ancestry (yellow).

Abbreviations: AFR, African; AMR, Ad Mixed American; EAS, East Asian; EUR, European; SAS, South Asian; NA, not available.

.

*
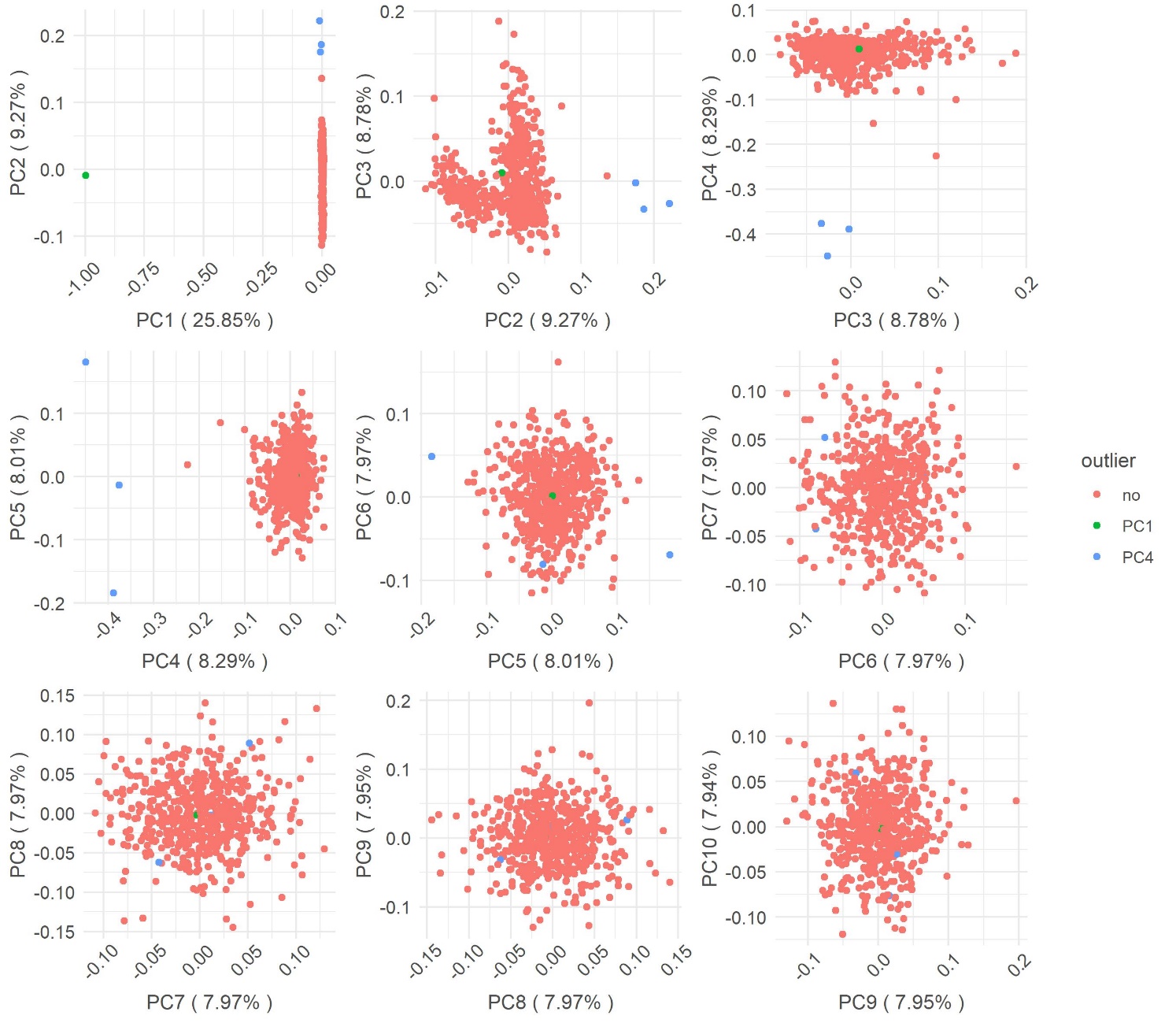
*

**Figure S2b | Pairwise-comparison plots of the first ten genetic principal components for cases and controls.**

Every dot represents a single individual and is colored according to the principal component it is outlying. In total, four samples are outliers according to the set criteria: one sample according to PC1 (green) and three samples according to PC4 (blue).

**
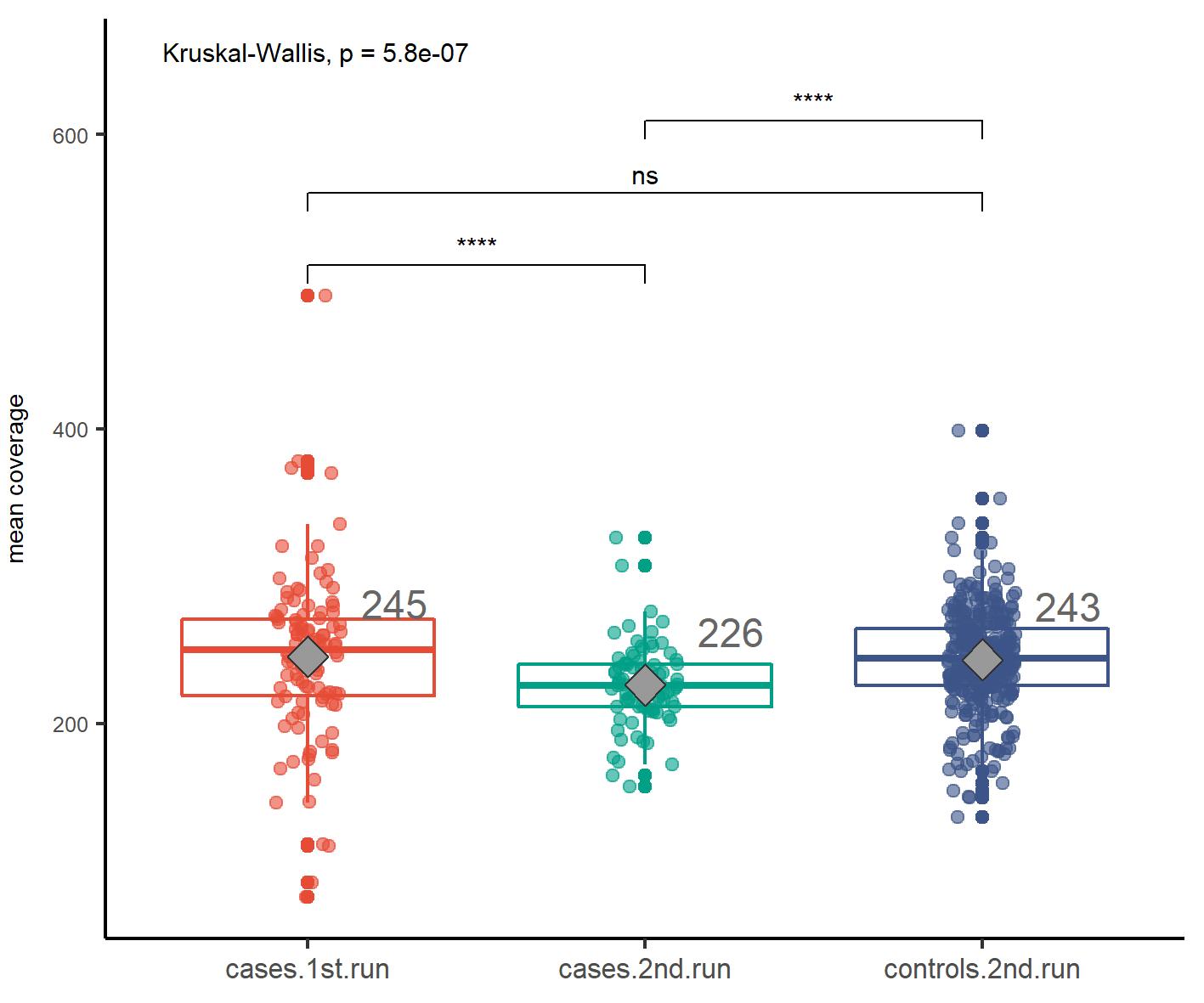
**

**Figure S3 | Mean coverages of 1^st^ run cases and 2^nd^ run cases and controls.**

Each dot represents a single individual stratified by case/ control status and the run in which the sample was sequenced. The mean coverage per group is annotated in the plot and depicted by the grey diamond.

Supplementary Tables

**Table S1 | Overview of pathogenic HAE variants in the sequenced genes.**

| Gene | OMIM  Phenotype | HAE-associated nucleotide change | Amino acid  change | Initial description |
| --- | --- | --- | --- | --- |
| *SERPING1* | 106100 | numerous* | numerous* | Stoppa-Lyonnet et al., 1987 |
| *F12* | [610618](https://www.omim.org/entry/610618) | c.983C>A | p.T328K | Cichon et al., 2006;  Dewald and Bork, 2006 |
|  |  | c.983C>G | p.T328R | Cichon et al., 2006;  Dewald and Bork, 2006 |
|  |  | c.971_1018+24del72 | indel | Bork et al., 2014 |
|  |  | c.892_909dup | p.P298_P303dup | Kiss et al., 2013 |
| *PLG* | [619360](https://www.omim.org/entry/619360) | c.988A>G | p.K330E | Bork et al., 2018 |
| *ANGPT1* | [619361](https://www.omim.org/entry/619361) | c.807G>T | p.A119S | Bafunno et al., 2018 |
| *KNG1* | [619363](https://www.omim.org/entry/619363) | c.1136T>A | p.M379K | Bork et al., 2019 |

* until to date, over 700 causative HAE mutations have been identified in the *SERPING1* gene. For a comprehensive overview we refer to the review by Ponard et al., 2020.

Abbreviations: HAE, hereditary angioedema; OMIM, Online Mendelian Inheritance in Men.

**Table S2 | SmMIP sequences.**

| Primer Sequence | Primer Name | | |
| --- | --- | --- | --- |
| GTGGAGACGGGAGGCGGGATGGTGCGGCTTCAGCTTCCCGATATCCGACGGTAGTGTNNNNNAGGGTGGGAGCTGGCT | SERPING1_001 | | |
| ACTACGAGGCACAGTCCTCTAACTTCAGCTTCCCGATATCCGACGGTAGTGTNNNNNCCTCCAGGATGGGTTCAACGAAT | SERPING1_002 | | |
| GACCTTCCCTTCGCCTCTGTCTTGCAAACCTTCAGCTTCCCGATATCCGACGGTAGTGTNNNNNGTGGGTTGGGTGGTGG | SERPING1_003 | | |
| GCTCTGTGGTGGGTTGTGTGGTGGCTTCAGCTTCCCGATATCCGACGGTAGTGTNNNNNAAATCTACCAAAGCATCC | SERPING1_004 | | |
| CCCAACACGGCCTCTGTTGAATGACTCTCCTTCAGCTTCCCGATATCCGACGGTAGTGTNNNNNCCAACAAATGACCTGG | SERPING1_005 | | |
| CACGACCAAAGGTGTCACCTCAGTCTCTCTTCAGCTTCCCGATATCCGACGGTAGTGTNNNNNTCCCCAACCCTCATTC | SERPING1_006 | | |
| GTCCTGAGAGGACTCTGAAGGGGGACCCACTTCAGCTTCCCGATATCCGACGGTAGTGTNNNNNAACACCAAAACAAACC | SERPING1_007 | | |
| GTCCTGAACACGTCGTTCTTTCCATGAGCCTTCAGCTTCCCGATATCCGACGGTAGTGTNNNNNGTTGTTGGTGTTCTTG | SERPING1_008 | | |
| GTTGGCGTCACTGTTGTTGCTTAGGACTCTTCAGCTTCCCGATATCCGACGGTAGTGTNNNNNAAGAAGGACCCAGAATG | SERPING1_009 | | |
| GGAAGAGGTGGGAGGGTTGCTTCAGCTTCCCGATATCCGACGGTAGTGTNNNNNGGAGAAGGAAAGGTTAAGAAC | SERPING1_010 | | |
| GTCAATGAAATGGGCCACAGGGTCTTCAGCTTCCCGATATCCGACGGTAGTGTNNNNNAGGAGAAAAGATAGGGTGG | SERPING1_011 | | |
| GCCATCATGGAGAAACTGGAGATGTCCACTTCAGCTTCCCGATATCCGACGGTAGTGTNNNNNAGGAGAGAGATGCGGT | SERPING1_012 | | |
| CAGAGGAGAAAGGGGGGATCCCTAAGATGCTTCAGCTTCCCGATATCCGACGGTAGTGTNNNNNCCCTTCTGTTTTCAAG | SERPING1_013 | | |
| GCAGGGTGCGGGCCACAGAGATGGCGGCTTCAGCTTCCCGATATCCGACGGTAGTGTNNNNNTGAGGCTGGAGAGGTAG | SERPING1_014 | | |
| GCATCGCAGAAACCTGAAGATCTGGGTCTTCAGCTTCCCGATATCCGACGGTAGTGTNNNNNCCATGAAGACAGGGAA | SERPING1_015 | | |
| CAGAGTCAGAAGCCAGCATGATACCCTCACTTCAGCTTCCCGATATCCGACGGTAGTGTNNNNNGAGATGGCGGAGGCTG | SERPING1_016 | | |
| GTTTCCGACCCAGGGTGATTCCGGAGGCCCTTCAGCTTCCCGATATCCGACGGTAGTGTNNNNNATCCTCCATCCTCCCC | F12_001 | | |
| GCGTGCCAGGTGAGCTCTTAGCCCGGTTCTTCAGCTTCCCGATATCCGACGGTAGTGTNNNNNTGGGGTGTGAAGAAGG | F12_002 | | |
| GCAACAAGCCAGGCGTCTACACCGATGTGCTTCAGCTTCCCGATATCCGACGGTAGTGTNNNNNCAAATCTCAGGTCCAC | F12_003 | | |
| GTGATTCCGCAGTGAGAGAGTGGCTCTTCAGCTTCCCGATATCCGACGGTAGTGTNNNNNGCTGGTGTGTGAGGAC | F12_004 | | |
| GAATGGGTGGCGCTGACCTGATGGGTTGTCTTCAGCTTCCCGATATCCGACGGTAGTGTNNNNNCACGCTCTGCCAGGTG | F12_005 | | |
| CTGCCGTCCGCATCCTCCCTTCAGCTTCCCGATATCCGACGGTAGTGTNNNNNCTTCTTCCGCCTAACCCAGTGATCA | F12_006 | | |
| CCGGGGCCCCAAGCTCTCTTCTTCAGCTTCCCGATATCCGACGGTAGTGTNNNNNTGGCAGAGCGTGGTCTCGGAGGGT | F12_007 | | |
| GCGGTAGGAGCGCACGGCCAACGTCTGGCTTCAGCTTCCCGATATCCGACGGTAGTGTNNNNNTGAAGGCGCAACAGAG | F12_008 | | |
| GCCACAAGCGTTCTGGGGAAGCTTCAGCTTCCCGATATCCGACGGTAGTGTNNNNNTGCTGGTAGCTGACGGGCGAG | F12_009 | | |
| GCCAACGACGCGGGTCATCGAAGACAGACCTTCAGCTTCCCGATATCCGACGGTAGTGTNNNNNCTGGGGCGGCTCTGGG | F12_010 | | |
| GCACCCCTACATCGCCGCGCTGTACTCTTCAGCTTCCCGATATCCGACGGTAGTGTNNNNNCTGCTCCTCCACAGCC | F12_011 | | |
| GCGGCTCCGCAAGAGTCTGTCTTCGATGACTTCAGCTTCCCGATATCCGACGGTAGTGTNNNNNAGGAAGTGGGGGGGGG | F12_012 | | |
| GCTGCGCGGGCATGAGTGGGACATGAAGCTTCAGCTTCCCGATATCCGACGGTAGTGTNNNNNGGCAAGGCTGTGGAGG | F12_013 | | |
| GCCTGGGTTGGGGTCTGGCACTGTCTTCAGCTTCCCGATATCCGACGGTAGTGTNNNNNCTCTCGGCTCCTCCTT | F12_014 | | |
| GCCACACGACGGGGCGCCGTTAGAGCTTCAGCTTCCCGATATCCGACGGTAGTGTNNNNNGGCATGAGTGGGACATG | F12_015 | | |
| GCTGGGAGTACTGCGACCTGGCACACTTCAGCTTCCCGATATCCGACGGTAGTGTNNNNNGGAACTGGGGACTGGG | F12_016 | | |
| GCCTTGGTGTCTGAGGAGAAAGGGGCTTCAGCTTCCCGATATCCGACGGTAGTGTNNNNNGGCAGAAGGCGTGGCC | F12_017 | | |
| TGATGGCCGCGGGCTCAGCTACCTTCAGCTTCCCGATATCCGACGGTAGTGTNNNNNGTGGGTGAGTGAGGGTCTGGGG | F12_018 | | |
| SCCTTCTGCGACGTGGGTGAGTGAGGCTTCAGCTTCCCGATATCCGACGGTAGTGTNNNNNGGAGAGCTCTCTGGGG | F12_019_SNP | | |
| GATGAGAGGGAGGCAGGAGAGCCCACTTCAGCTTCCCGATATCCGACGGTAGTGTNNNNNTGCCTAGAGGTGGAGGG | F12_020 | | |
| GTTGGGAACGGGCCAGGGAGGAGCGTCACTTCAGCTTCCCGATATCCGACGGTAGTGTNNNNNTATCCCTCTTTGTCCC | F12_021 | | |
| GGTATAGAACTGAGCAAGCAGCCTTCAGCTTCCCGATATCCGACGGTAGTGTNNNNNTGGGCGGGGTGCTGGGGG | F12_022 | | |
| GCTGCAGTGGTCTGAGAGATGGACATGGTCTTCAGCTTCCCGATATCCGACGGTAGTGTNNNNNTGTAGGCCCAGGGTTG | F12_023 | | |
| GGGACCACTCCTTCCCAGAACTCTCCCTCTTCAGCTTCCCGATATCCGACGGTAGTGTNNNNNGGCAGGGGCTGTGTTT | F12_024 | | |
| GAGACAAGGCTTCCCTGCTCTACCCAGCTTCAGCTTCCCGATATCCGACGGTAGTGTNNNNNGGTGTGTGGGGTCTGG | F12_025 | | |
| GCCAGGCCCTCAGCCCTGGTAAGACTACTTCAGCTTCCCGATATCCGACGGTAGTGTNNNNNTTTTCCTGACCAGACC | F12_026 | | |
| GGGTAGAGCAGGGAAGCCTTGTCTCTTTCTTCAGCTTCCCGATATCCGACGGTAGTGTNNNNNACAAATGTACCCACAA | F12_027 | | |
| GTCTAGTCTAGTGCCTACCTGGTGCTAGGCTTCAGCTTCCCGATATCCGACGGTAGTGTNNNNNTTCCCTGCCTTCTTCT | F12_028 | | |
| GCTGTGGGAACCAGGATTGTCCCAGGATTCTTCAGCTTCCCGATATCCGACGGTAGTGTNNNNNTCAGGAGGGCAGCTTG | F12_029 | | |
| GTTTGTGGGTGGGGGTGAAATGAAGAGCTCTTCAGCTTCCCGATATCCGACGGTAGTGTNNNNNCAGATCAATAGGACTG | F12_ERE_030 | | |
| CAATCTCCCTCTAGGAGCTGAGGGCTTCAGCTTCCCGATATCCGACGGTAGTGTNNNNNCTGGTTGTTACTTTGGTTTTG | KNG1_001 | | |
| GCATCCACAGCTTTAAATAAATCCTTCAGCTTCCCGATATCCGACGGTAGTGTNNNNNGGTGGTATGTGTGTGTGT | KNG1_002 | | |
| GTATTGGCCATTCTTGGGCCTTCTGTYTTCTTCAGCTTCCCGATATCCGACGGTAGTGTNNNNNTGGGTCAGTTGGATGA | KNG1_003_SNP | | |
| GCCAGGAACACAATCTTGACCAGGCTCTCTTCAGCTTCCCGATATCCGACGGTAGTGTNNNNNTGATCTCTTTCTTTTCT | KNG1_004 | | |
| GATCGCAATSAGCATTCGCATACACTGCCCTTCAGCTTCCCGATATCCGACGGTAGTGTNNNNNTGTTGAGTGTTGTTGT | KNG1_005_SNP | | |
| GCCGTGTCTCAGAATGGGCTCCCTTCAGCTTCCCGATATCCGACGGTAGTGTNNNNNAAAACACCACCAGCCATGCAA | KNG1_006 | | |
| GTCCCTTTGGAATGGTGTAAGTAGGCTTCAGCTTCCCGATATCCGACGGTAGTGTNNNNNCAGCGAATAATGTTTAAAC | KNG1_007 | | |
| GCTGGGTGGGAAGACTGTCACGAAAAGTCTTCAGCTTCCCGATATCCGACGGTAGTGTNNNNNCTCAATTGTGCAAACGA | KNG1_008 | | |
| GGTCCCAGACAACTGGCTGAGTCTTTTCCCTTCAGCTTCCCGATATCCGACGGTAGTGTNNNNNTTTAACTGAGCACTTA | KNG1_009 | | |
| GAAGTAGCAGCCTGGCCTAGCATCCCTTCAGCTTCCCGATATCCGACGGTAGTGTNNNNNGTTGCGTTATTCTCTGCATT | KNG1_010 | |  |
| GCAGCCCACGCAAATCTTGGTAGGTGGTTCTTCAGCTTCCCGATATCCGACGGTAGTGTNNNNNGTGTTACTGCTTTTGT | KNG1_011 | |  |
| CATGCACCTGTCTACTTTTTCACTGGAAGCTTCAGCTTCCCGATATCCGACGGTAGTGTNNNNNGCAAATATTTTTAAGC | KNG1_012 | |  |
| GCTCATTCTGAAAATCCATATTTGGGGGCTTCAGCTTCCCGATATCCGACGGTAGTGTNNNNNAACACTGTCTCTCTTTC | KNG1_013 | |  |
| GTATTACTGCAAAAATCATGCTATTGATGCTTCAGCTTCCCGATATCCGACGGTAGTGTNNNNNCCTTGTTCTTTTCCTG | KNG1_014 | |  |
| GCAGGTGCCATGGAAGTGTGGGGTCTTCAGCTTCCCGATATCCGACGGTAGTGTNNNNNGGCCATGTCCTCTTTGGTG | KNG1_015 | |  |
| GCCCTTGGTCACGTTCATGTTTATGGCCTTCAGCTTCCCGATATCCGACGGTAGTGTNNNNNCATTCTTTTTGCCTTT | KNG1_016 | |  |
| GTTTTCCGTGGCCATGACCATGCTCTTCAGCTTCCCGATATCCGACGGTAGTGTNNNNNTGGCTTGGCTAGGGAAGGG | KNG1_017 | |  |
| GTCTTCTCTTGTGTCTGTGCAGAAGGTGTCTTCAGCTTCCCGATATCCGACGGTAGTGTNNNNNTTGGGTCTATCTGGAT | KNG1_018 | |  |
| GTRTGGGAGCTGGTGATATAGGAGGCATCCTTCAGCTTCCCGATATCCGACGGTAGTGTNNNNNTTGTGTGGTTGGATTA | KNG1_019_SNP | |  |
| GGAGGTCGTGTCTGGAAAATCTGATATTGCTTCAGCTTCCCGATATCCGACGGTAGTGTNNNNNGGAGAGAGGGATATTG | KNG1_020 | |  |
| GTATAGTAAAACGCAGTTAATATGATGTCTTCAGCTTCCCGATATCCGACGGTAGTGTNNNNNTCTTGTCCTTCCTCTC | KNG1_021 | |  |
| ACATTCCATTTAGATTGGAGGGGCCACCTTCAGCTTCCCGATATCCGACGGTAGTGTNNNNNCTCCGGATTTCTTTGTTG | ANGPT1_001 | |  |
| CTTGCTTGTTCTTGTTATGCTTATTTGCACTTCAGCTTCCCGATATCCGACGGTAGTGTNNNNNTGGTTTTGTCCCGCAG | ANGPT1_002 | |  |
| ATTGGTTTGGGGCTTAAGGTTTCTTATCTCTTCAGCTTCCCGATATCCGACGGTAGTGTNNNNNTTTAAAAGGTCACACT | ANGPT1_003 | |  |
| GCAAATGTGCCCTCATGTTAACAGGAGGTCTTCAGCTTCCCGATATCCGACGGTAGTGTNNNNNTGGGTTCTGTTATTCT | ANGPT1_004 | |  |
| GTGTAGTGTTCGACTACCTTTTACCTAGCCTTCAGCTTCCCGATATCCGACGGTAGTGTNNNNNGAATATTGGCTGGGGR | ANGPT1_005_SNP | |  |
| GGAAGGGAACCGAGCCTATTCACAGTATGCTTCAGCTTCCCGATATCCGACGGTAGTGTNNNNNGCTGAACATGAAAAGA | ANGPT1_006 | |  |
| GSAAGAATTTATGGTGCTTTTTGGTGTTCTTCAGCTTCCCGATATCCGACGGTAGTGTNNNNNATGTTTTTTAAAGTAG | ANGPT1_007_SNP | |  |
| GCCAGATCCCAGTTGAATTGCTGGACTTCAGCTTCCCGATATCCGACGGTAGTGTNNNNNGCAGTTTTACTAAAGGGAGG | ANGPT1_008 | |  |
| GCAGATGTATATCAAGCTGGTTCTTCAGCTTCCCGATATCCGACGGTAGTGTNNNNNGTGCGATATTGACATTTGTGTGG | ANGPT1_009 | |  |
| ATGTCTTCCTCACTTTGGTATTGTTAATACTTCAGCTTCCCGATATCCGACGGTAGTGTNNNNNCCTTCAAGGCTTGGTT | ANGPT1_010 | |  |
| CAGAGCTACCACCAACAACAGTGTCCTTCCTTCAGCTTCCCGATATCCGACGGTAGTGTNNNNNGTTTTATTTTCACTTC | ANGPT1_011 |  |  |
| GTTTCTGTGTGTACTTATTTATATCCTTCCTTCAGCTTCCCGATATCCGACGGTAGTGTNNNNNAATCAAACTTCTCGAC | ANGPT1_012 |  |  |
| GAGATACAGCTGCTGGAGAATTCCTTCAGCTTCCCGATATCCGACGGTAGTGTNNNNNTGCGCCCGGCGGTAAACCATTT | ANGPT1_013 |  |  |
| CGACTTCATGTTTTCCACAATGTAATTCCTTCAGCTTCCCGATATCCGACGGTAGTGTNNNNNTTCCTTGTTGAGTCTG | ANGPT1_014 |  |  |
| AGACACCGCTGGCAAATCAGCCATCTCTTCAGCTTCCCGATATCCGACGGTAGTGTNNNNNGGTAGCCGTGTGGTTCTG | ANGPT1_015 |  |  |
| GCACGGACCTTTTTCCTTTCTTGCACTGCTTCAGCTTCCCGATATCCGACGGTAGTGTNNNNNCGTGAGAGTACGACAGA | ANGPT1_016 |  |  |
| CAACACAAACGCTCTGCACTTCAGCTTCCCGATATCCGACGGTAGTGTNNNNNTTTCCTTTGCTTTCCTCGCTGCCATTC | ANGPT1_017 |  |  |
| GACTCACATAGGGTGCAGCAATCAGCGCCCTTCAGCTTCCCGATATCCGACGGTAGTGTNNNNNTTTGAGGGGGAAAGAG | ANGPT1_018 |  |  |
| CATTTAATTTTTGATTCATGAAACTTCCCTTCAGCTTCCCGATATCCGACGGTAGTGTNNNNNTCTGGGCACTGCTGGC | PLG_001 |  |  |
| GCAAAATGTGAGGAGGACGAAGAATTCACCTTCAGCTTCCCGATATCCGACGGTAGTGTNNNNNTTTACTGACCATTTAT | PLG_002 |  |  |
| GAGGAAAGAGAAATTTATGGAGCCAGAGTCTTCAGCTTCCCGATATCCGACGGTAGTGTNNNNNGGGAGCAGGAAGTATA | PLG_003 |  |  |
| TCCTCCCCATCCTCCCRCTCCTTCAGCTTCCCGATATCCGACGGTAGTGTNNNNNTACTTATTGGATTTCCTGCTTCGTT | PLG_004_SNP |  |  |
| CACAAGACACCACATGAAGGTCTGCACAGCTTCAGCTTCCCGATATCCGACGGTAGTGTNNNNNGTGGGGAGAAGTGGAA | PLG_005 |  |  |
| GTARTTCTTTCCATTCCCAGTCTTCTTCAGCTTCCCGATATCCGACGGTAGTGTNNNNNAACCAATCCCTCACAGACACA | PLG_006_SNP |  |  |
| GCGACATTCTTGAGTGTGAAGGTCTTCAGCTTCCCGATATCCGACGGTAGTGTNNNNNGCTGATTTTTAGAATATAGTCT | PLG_007 |  |  |
| GCCCCTTCCCACAGGGATGTTATTACTTCAGCTTCCCGATATCCGACGGTAGTGTNNNNNAACTACTGCAGGAATCCAGA | PLG_008 |  |  |
| ACATTCCATGTTTAATTAAGGCTCTGCCTTCAGCTTCCCGATATCCGACGGTAGTGTNNNNNTCCTTCCTTCCCACTCT | PLG_009 |  |  |
| GTCAGTGCCTGAGTGCAGCCTCTGCTTCAGCTTCCCGATATCCGACGGTAGTGTNNNNNAGAATTACTGTCGTAACCCCG | PLG_010 |  |  |
| GATTCCAGGATTTGGACCTGCCCTGTTCTCTTCAGCTTCCCGATATCCGACGGTAGTGTNNNNNAAAATCTTKCTTGTCC | PLG_011_SNP |  |  |
| GACCCCTCACACACATAACACTTCAGCTTCCCGATATCCGACGGTAGTGTNNNNNCTCCGTCTCAAAAAATATATATATT | PLG_012 |  |  |
| CRGTCTCATTCTGCTGCTATGGAATGTGACTTCAGCTTCCCGATATCCGACGGTAGTGTNNNNNTCTTCTGGTCCCACCT | PLG_013_SNP |  |  |
| CAAAAAGAAAAAAGTCTAGGGAACCACGCCTTCAGCTTCCCGATATCCGACGGTAGTGTNNNNNTCCGTGGATACTGGGG | PLG_014 |  |  |
| GCACTTGGCTGTTGGTTGTATGGCACCACTTCAGCTTCCCGATATCCGACGGTAGTGTNNNNNTTACAAAGCTACTGTA | PLG_015 |  |  |
| CAATTACTGAAAAAAAAGAAGCATGAAGCCTTCAGCTTCCCGATATCCGACGGTAGTGTNNNNNTGCCGGTGTGGTGTCA | PLG_016 |  |  |
| GTGGTGGTGGTGGAGGATGTCTTCAGCTTCCCGATATCCGACGGTAGTGTNNNNNGTCCTAGGAAGTTGGCTTGAAG | PLG_017 |  |  |
| CGAGTGTTGTAGCACCTCCGCCTGTTGTCCTTCAGCTTCCCGATATCCGACGGTAGTGTNNNNNTTCTCCCACCTCTTGT | PLG_018 |  |  |
| GCAGAAACCTTCCATGCTACACGAGAAACTTCAGCTTCCCGATATCCGACGGTAGTGTNNNNNAACCTGAAAAAATGCTC | PLG_019 |  |  |
| ACAGAGACCCAGGATGATATGGAATCCTTCAGCTTCCCGATATCCGACGGTAGTGTNNNNNGTGGATTTGTCTCTGG | PLG_020 |  |  |
| GTCTATGGGGCTCCTGGGCAGCCCAGTCCTTCAGCTTCCCGATATCCGACGGTAGTGTNNNNNCCCCCATTACAAAAAA | PLG_021 |  |  |
| GAGGGAGAAGGTGTTCCAAGGCTCACRCACTTCAGCTTCCCGATATCCGACGGTAGTGTNNNNNTACCAAAAAGAAGGCA | PLG_022_SNP |  |  |
| GAAACGATTTATACTGTCCCTCCACGTAACTTCAGCTTCCCGATATCCGACGGTAGTGTNNNNNATTTCTTTCCCACCTT | PLG_023 |  |  |
| GTTAAATTGATACTTTGTTCTGCTCCATTCTTCAGCTTCCCGATATCCGACGGTAGTGTNNNNNAGAAGGACAGAAAAAG | PLG_024 |  |  |
| YGTCAAGAGGAAAATATGGTCCAGCCCCTCTTCAGCTTCCCGATATCCGACGGTAGTGTNNNNNGTACCTGCTTAGCTTT | PLG_025_SNP |  |  |
| GTGTGCACCCAGGATGACCTTGTAGGATGCTTCAGCTTCCCGATATCCGACGGTAGTGTNNNNNAAACCCAGACATAAAG | PLG_026 |  |  |
| GCATCAGCAGTTATGTTTGACTGCTCTGCTTCAGCTTCCCGATATCCGACGGTAGTGTNNNNNAGGTCAAAACCAATTC | PLG_027 |  |  |
| GGCAACTGCACCCAAAACACCTTCAGCTTCCCGATATCCGACGGTAGTGTNNNNNTGGCAACTGTCAGTGCCTCCGGC | PLG_028 |  |  |
| GCACAGAGTTCGGTGGATTGGACTCTTCTTCAGCTTCCCGATATCCGACGGTAGTGTNNNNNTAAACAACACTTAGAC | PLG_029 |  |  |
| GTTAGGCTGCCTGCCTTTTATTATGGGATCTTCAGCTTCCCGATATCCGACGGTAGTGTNNNNNAACAAACCTTGAAACA | PLG_030 |  |  |
| ACCAGAGGMCCTCCACTGTCACCCTATACCTTCAGCTTCCCGATATCCGACGGTAGTGTNNNNNCGTCACTCTGTCTCCC | PLG_031_SNP |  |  |

Note: Known variants (MAF > 1%) within the sequence of the ligation or extension arm are encoded by ambiguous coding (highlighted in light blue) to obtain primers that comprise a mixture of reference and alternative allele(s) at the respective bp position. The NNNNN sequence included in each smMIP is representative of the five degenerate bases incorporated during smMIP synthesis.

**Table S3 | Sanger sequencing primers.**

| Primer Sequence | Primer Name |
| --- | --- |
| CCACACCTTCTCTTCCTGCT | SERPING1_Ex3_F |
| CCAGAGGCATGGCTTTGTAA | SERPING1_Ex3_R |
| ACGTGACTGCCGAGCAAG | F12_Ex9_F |
| CCTCTCGGCTCCTCCTTC | F12_Ex9_R |

**References**

Bafunno, V., Firinu, D., D’Apolito, M., Cordisco, G., Loffredo, S., Leccese, A., et al. (2018). Mutation of the angiopoietin-1 gene (ANGPT1) associates with a new type of hereditary angioedema. *Journal of Allergy and Clinical Immunology*. doi: 10.1016/j.jaci.2017.05.020.

Bork, K., Wulff, K., Hardt, J., Witzke, G., and Lohse, P. (2014). Characterization of a partial exon 9/intron 9 deletion in the coagulation factor XII gene (F12) detected in two Turkish families with hereditary angioedema and normal C1 inhibitor. *Haemophilia* 20, e372–e375. doi: 10.1111/hae.12519.

Bork, K., Wulff, K., Rossmann, H., Steinmüller‐Magin, L., Brænne, I., Witzke, G., et al. (2019). Hereditary angioedema cosegregating with a novel kininogen1 gene mutation changing the N‐terminal cleavage site of bradykinin. *Allergy*, all.13869. doi: 10.1111/all.13869.

Bork, K., Wulff, K., Steinmüller-Magin, L., Brænne, I., Staubach-Renz, P., Witzke, G., et al. (2018). Hereditary angioedema with a mutation in the plasminogen gene. *Allergy: European Journal of Allergy and Clinical Immunology*. doi: 10.1111/all.13270.

Cichon, S., Martin, L., Hennies, H. C., Müller, F., Driessche, K. van, Karpushova, A., et al. (2006). Increased Activity of Coagulation Factor XII (Hageman Factor) Causes Hereditary Angioedema Type III. Available at: www.ajhg.org.

Dewald, G., and Bork, K. (2006). Missense mutations in the coagulation factor XII (Hageman factor) gene in hereditary angioedema with normal C1 inhibitor. *Biochemical and Biophysical Research Communications* 343, 1286–1289. doi: 10.1016/j.bbrc.2006.03.092.

Kiss, N., Barabás, E., Várnai, K., Halász, A., Varga, L. Á., Prohászka, Z., et al. (2013). Novel duplication in the F12 gene in a patient with recurrent angioedema. *Clinical Immunology* 149, 142–145. doi: 10.1016/j.clim.2013.08.001.

Ponard, D., Gaboriaud, C., Charignon, D., Ghannam, A., Wagenaar-Bos, I. G. A., Roem, D., et al. (2020). SERPING1 mutation update: Mutation spectrum and C1 Inhibitor phenotypes. *Human Mutation* 41, 38–57. doi: 10.1002/humu.23917.

Stoppa-Lyonnet, D., Tosi, M., Laurent, J., Sobel, A., Lagrue, G., and Meo, T. (1987). Altered C1 Inhibitor Genes in Type I Hereditary Angioedema. *New England Journal of Medicine* 317, 1–6. doi: 10.1056/NEJM198707023170101.
